# Wettability of Sn-3.0Ag-0.5Cu Solder Reinforced with TiO_2_ and Al_2_O_3_ Nanoparticles at Different Reflow Times

**DOI:** 10.3390/nano13202811

**Published:** 2023-10-23

**Authors:** Nur Haslinda Mohamed Muzni, Ervina Efzan Mhd Noor, Mohd Mustafa Al Bakri Abdullah

**Affiliations:** 1 Faculty of Engineering and Technology, Multimedia University, Ayer Keroh 75450, Malacca, Malaysia; 2 Faculty of Chemical Engineering and Technology, Universiti Malaysia Perlis, Arau 02600, Perlis, Malaysia

**Keywords:** nanoparticles, wettability, nanocomposite

## Abstract

This study investigated the influence of reinforcing 0.50 wt.% of titanium oxide (TiO_2_) and aluminium oxide (Al_2_O_3_) nanoparticles on the wettability performance of a Sn-3.0Ag-0.5Cu (SAC305) solder alloy. The thermal properties of the SAC305 nanocomposite solder are comparable with thos of an SAC305 solder with a peak temperature window within a range of 240 to 250 °C. The wetting behaviour of the non-reinforced and reinforced SAC305 nanocomposite solder was determined and measured using the contact angle and spreading area and the relationships between them were studied. There is an increment in the spreading area (5.6 to 7.32 mm) by 30.71% and a reduction in the contact angle (26.3 to 18.6°) by 14.29% with an increasing reflow time up to 60 s when reinforcing SAC305 solder with 0.50 wt.% of TiO_2_ and Al_2_O_3_ nanoparticles. The SAC305 nanocomposite solder has a better wetting performance compared with the SAC305 solder. As the reflow time increased, the spreading area increased and the contact angle decreased, which restricted intermetallic compound growth and thus improved wettability performance

## 1. Introduction

Soldering is a technique of metallurgical bonding used for joining two types of metals together, through the melting of an alloy metal known as solder. A solder is a metal alloy, usually made up from tin and lead with a melting temperature of 183 °C, is melted to form a mechanical or electrical bond, commonly in the electronic packaging industry. The wettability or spreadability of the solder is essential to create a strong mechanical and electronic bond among two different metal surfaces.. The wettability or spreadability is the ability of solder to wet or spread easily over a metal surface and form metallurgical bonding [1,2]. The demands for the miniaturization of the electronic devices have urge the development of new or improved solder alloy for better solder joint performances. The new and advanced solder technology also contributes to replace the conventional tin–lead solder due to restriction of the use of hazardous substances which cause health and environmental concerns [3,4,5]. Thus, it is important to understand the wetting performance of newly developed solders to enhance the reliability of solder joints. 

In the process of development of new lead-free solders, there are several basic requirements that have to be fulfilled for them to be comparable with the conventional eutectic tin–lead solder. In terms of wettability, they should be able to reduce the surface tension of pure Sn. The formation of intermetallic compounds (IMCs) quickly occurs and prevents transformation of ß-Sn to α-Sn. Additionally, they should be capable to maintain the melting temperature at around 183 °C with a eutectic or near eutectic composition. Sn-Ag-Cu solder alloys have been investigated as promising solder alloys in replacing the toxic tin–lead solder alloy; apparently due to its good mechanical properties [6,7,8,9]. SAC solder alloys have higher melting points than tin–lead solders but possess good mechanical properties, higher strength, excellent thermal fatigue characteristics, and better creep properties [10,11,12,13]. Among the SAC solder family, SAC305 (Sn-3.0Ag-0.5Cu) is a lead-free alloy recommended for lead-free soldering by the JEIDA, that offers superior fluidity. Numerous research have been conducted to investigate the wetting behaviour of SAC solders on their solderability [8,14,15,16]. The presence of other elements in the solder materials has been found to enhance the wetting properties of solder alloys by reducing the surface tension, having lower contact angles, bigger spreading areas, and shorter wetting times [9,17,18,19].

Several methods have been developed to produce SAC305 solders and one promising method is by reinforcing the SAC305 solders with additional elements [20,21]. The overall properties of the SAC305 solders could be greatly improved by reinforcing them with the additional elements. The trends of miniaturization in the electronic packaging industry have forced the use of much smaller electronic components, which require much smaller solder joints. This miniaturization trend somehow demands better solder joint reliability in the SAC305 solders. Thus, several research on the effects of nanoparticle addition to the SAC305 solder alloy on the wetting behaviour have been carried out [8,22,23,24]. Parallel to that, introducing smaller particles such as nanoparticles as reinforcement into the SAC305 solders could also enhance the reliability of the solder joints. Introduction of nanoparticles into lead-free solder is used for strengthening the solder by dispersion of particles at the interface between the solder and substrate by acting as a diffusion barrier for the relevant elements in the joint [25,26,27,28]. The strengthening effect of the nanoparticles is able to refine the solder grain, restrict the growth of IMCs, and thus enhance the overall performance of a solder alloy [26,29]. The fabrication of nanocomposite lead-free solder to replace conventional solder in the electronic packaging industry has been studied and proven to improve the overall performance of solder joints, attributed to the small size of nanoparticles and their characteristics as surface-active materials [12,30].

Ceramic nanoparticle reinforcements such as titanium oxide (TiO_2_), aluminium oxide (Al_2_O_3_), silicon oxide (SiO_2_), lanthanum oxide (La_2_O_3_), zirconium oxide (ZrO_2_), and iron oxide (Fe_2_O_3_) can be used as reinforcing materials to produce SAC305 nanocomposite solders and enhance the overall performance of the solder [20,31,32,33,34]. These ceramic nanoparticle materials that act as reinforcing agents were found to have good characteristics and enhanced mechanical properties (e.g., hardness, tensile strength, and shear strength) of solder alloys. Fe_2_O_3_ nanoparticles were investigated and enhanced the wettability of a nanocomposite solder and restricted intermetallic compound growth [35,36]. Al_2_O_3_ nanoparticles can restrict the growth of IMC layers, reduce the grain size, and improve the reliability of solder joints [15,20,37]. Additionally, TiO_2_ nanoparticles could also inhibit the growth of IMC layers, have better wettability, strengthen the solder joints, and increase the hardness of the solder [9,34,38]. ZrO_2_ nanoparticles were found to restrict IMC growth and to possess higher yield strength and supreme tensile strength, but to form pores in the nanocomposite solder [21,29]. However, restriction of IMC growth by ZrO_2_ nanoparticles was relatively weak compared to TiO_2_ and Al_2_O_3_ nanoparticles [39].

Although lots of studies have been carried out on the effects of reinforcing SAC305 solders with TiO_2_ or Al_2_O_3_ nanoparticles, there is limited research regarding the effects of SAC305 solder reinforced with both TiO_2_ and Al_2_O_3_ nanoparticles. Thus, this paper aims to study the effect of reinforcing SAC305 solder with both TiO_2_ and Al_2_O_3_ nanoparticles to produce SAC305 nanocomposite solder on the wettability performances analysed at different reflow times.

## 2. Methodology

**Figure 1:**
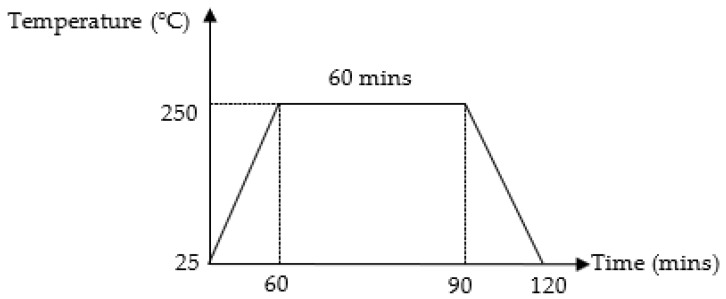
Typical reflow profile for preparing SAC305 nanocomposite solder.

SAC305 solder was prepared based on weight percentage (wt.%) of 96.5% tin (Sn), 3.0% argentum (Ag), and 0.5% copper (Cu). The SAC305 nanocomposite solder was prepared by adding 0.50 wt.% of TiO_2_ and Al_2_O_3_ nanoparticles in powder form with average sizes of 25–50 nm, respectively, into the lead-free SAC305 solder at 250 °C in a graphite crucible for 1 h. The nanocomposite solder was re-melted to obtain a homogeneous composition. The temperature profile for preparing the nanocomposite solder is shown in Figure 1. The nanocomposite solder alloy was then cut into 5 mm diameter billets with a thickness of 1 mm by using a puncher and later samples were cleaned with ethanol and rinsed with distilled water.

A sample from the nanocomposite solder alloy was taken to study the melting temperature by using differential scanning calorimetry (Mettler Toledo TGA/DSC, Columbus, OH, USA). The melting temperature was measured under argon atmosphere with a heating rate of 10 °C/min at a temperature ranging from 30 to 300 °C.

Commercial laminated copper plates with dimensions of 10 × 10 mm, 0.01 mm thick were used as substrates in this study for the wetting test. The SAC305 solder and the SAC305 nanocomposite solder alloy were reflowed for 20, 40, 60, and 80 s and the reflow temperature was fixed at 250 °C to form solder joints. A total of 6 samples was reflowed for each solder alloy at each reflow time to get average results.

The samples were then mounted in epoxy resin and cross-sectioned. The cross-sections were grinded by using silicon carbide sandpaper (grit P2000) to get flat surfaces. Following the grinding process, a polishing pad was used to polish the samples using 6, 3, and 1 µm diamond paste followed by alumina to observe the solder joints between the solder and the Cu substrate. A Kemet FORCIPOL 2V Grinder and Polisher machine (Kemet International Limited, Maidstone, UK) was used for grinding and polishing the samples.

**Figure 2:**
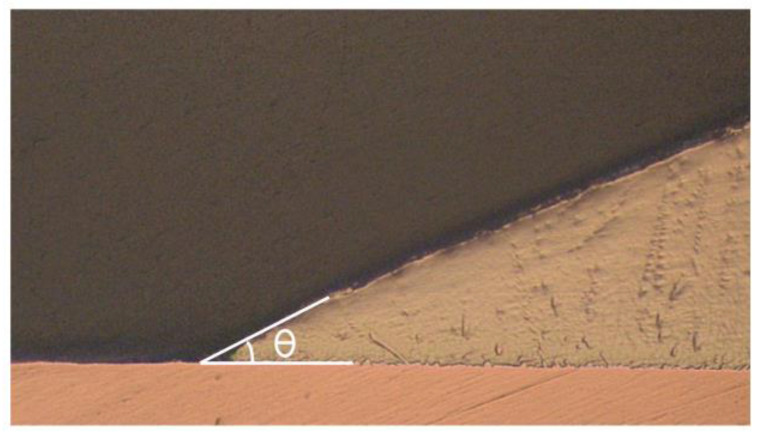
Contact angle (θ) of SAC305 nanocomposite solder on copper substrate.

**Figure 3:**
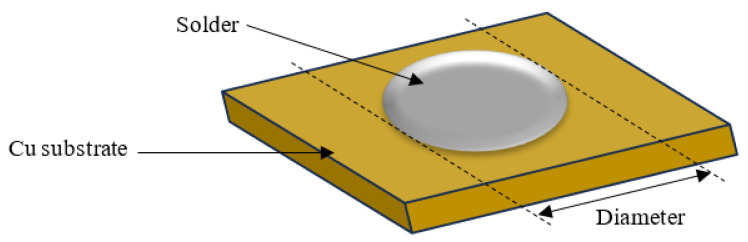
Schematic drawing of spreading test for SAC305 nanocomposite solder on copper substrate.

The wettability of the nanocomposite solders was evaluated by measuring the contact angles and spreading area. The contact angle was examined by using an optical microscope and measured with the aid of VIS Pro software version 3.30 (Figure 2) and the diameter of the solder was measured for the spreading area (Figure 3). The diameter of the solder on the Cu substrate was measured for the spreading area test. Average values for contact angles and spreading area were obtained. 

## 3. Results and Discussion

**Figure 4:**
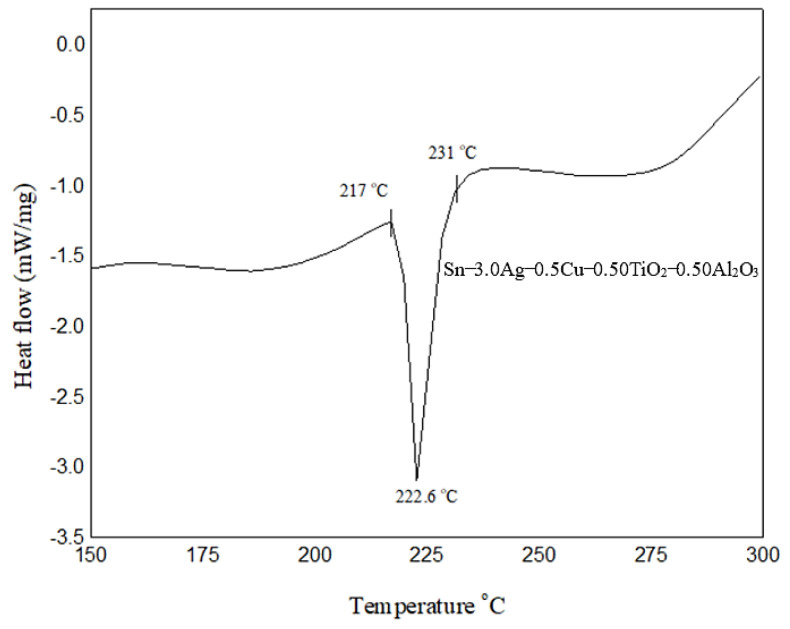
DSC analysis profile of the SAC305 nanocomposite solder alloy.

The results from DSC analysis are presented in Figure 4 for the SAC305 nanocomposite solder alloy. It can be interpreted from the result that the solidus temperature (onset) is 217 °C, the melting temperature (peak) is 222.6 °C, and the liquidus temperature is 231 °C, as these were recorded for SAC305 nanocomposite solder, as shown in Figure 4. Typically, SAC305 solder alloys begin to melt at 217 °C, reaching a fully liquid state at approximately 220 °C in the electronics industry. The typical peak reflow temperature target range for SAC305 solder alloys is between 240 and 250 °C. From the result shown, the solidus temperature for the SAC305 nanocomposite solder was similar to that of the SAC305 solder, which is 217 °C. In the meantime, the liquidus temperature of the SAC305 nanocomposite solder increased slightly to 231 °C as compared to the typical SAC305 solder. The slight increase in the heating profile was similarly found by [38,40]; the melting point of SAC305 solder increased slightly when it was reinforced with TiO_2_ and Al_2_O_3_ nanoparticles. The slight increment in the melting temperature was due to the high melting points of TiO_2_ (T_M_ = 1843 °C) and Al_2_O_3_ (T_M_ = 2072 °C) nanoparticles. The reason behind the high melting point is the local dissolution of TiO_2_ and Al_2_O_3_ nanoparticles [41]. Although there is a slight increment in the melting temperature of the solder, it is not a major concern as it still falls under the typical reflow temperature range for Pb-free solder, which is 240–250 °C.

**Figure 5:**
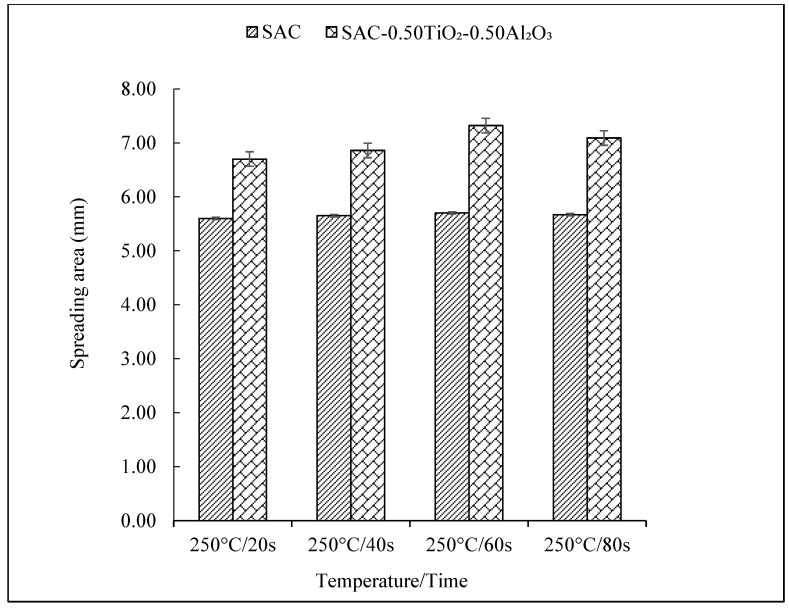
Spreading area of non-reinforced SAC305 and reinforced SAC305 nanocomposite solder at different reflow times.

**Figure 6:**
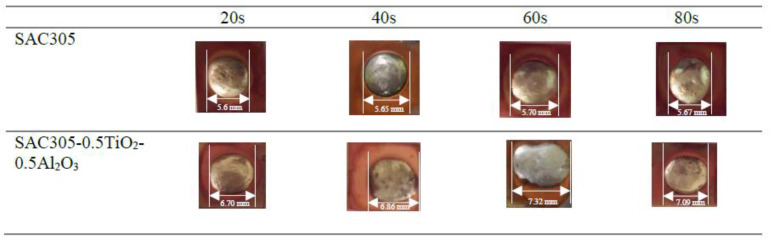
Top view images of the spreading area of non-reinforced SAC305 solder and reinforced SAC305 nanocomposite solder after reflow time.

**Figure 7:**
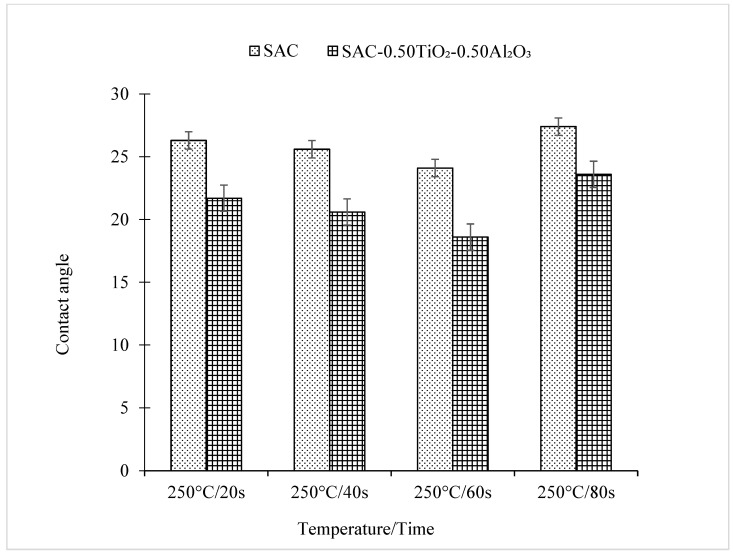
Contact angle of non-reinforced SAC305 and reinforced SAC305 nanocomposite solder at different reflow times.

**Figure 8:**
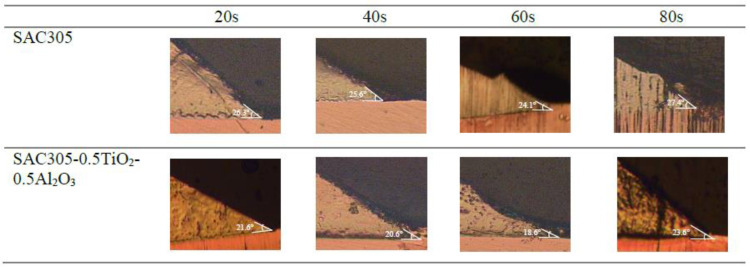
Cross-sectional view images on the contact angles of non-reinforced SAC305 and reinforced SAC305 nanocomposite solder at different reflow times.

The wettability of the non-reinforced and reinforced SAC305 nanocomposite solder was determined by observing the spreading area (Figure 5 and Figure 6), and the contact angle (Figure 7 and Figure 8) of the solder on Cu substrate was evaluated as a function of reflow time. As interpreted from the results shown, there was a significant increase in the spreading area of the solder on the Cu substrate as the reflow time increased from 20 s to 60 s as shown in Figure 5 and Figure 6. As compared with the non-reinforced SAC305 solder, reinforced SAC305 nanocomposite solders have much larger spreading areas after reinforcement with TiO_2_ and Al_2_O_3_ nanoparticles. The spread area of the SAC305 nanocomposite solder first increased significantly when it reflowed up to 60 s. By reinforcing it with 0.50 wt.% TiO_2_ and Al_2_O_3_ nanoparticles and reflowing for 60 s, the spread area can be increased by about 30.71% as compared with non-reinforced SAC305 nanocomposite solder: from 5.6 to 7.32 mm. An investigation by [37] also found a significant increase in the spreading area, by 15–40%, of SAC307 with incorporated 0.01 to 0.5 wt.% of Al_2_O_3_ nanoparticles. Then, the increment in the spread area decreased slightly with an increasing reflow time of 80 s. The reduction in the spreading area for the SAC305 nanocomposite solder reflowed for 80 s is about 3.14%. However, this reduction in spreading area for SAC305 nanocomposite solder (7.09 mm) is still better than that of non-reinforced SAC solder (5.67 mm). From these results, reinforcing the SAC305 solder with TiO_2_ and Al_2_O_3_ nanoparticles could remarkably improve the spreadability of the SAC305 solder.

Meanwhile, as the reflow time increased, an inverse trend in the contact angle between the solder and the Cu substrate was observed for both non-reinforced SAC305 solder and reinforced SAC305 nanocomposite solder, as shown in Figure 7 and Figure 8. The reinforced SAC305 nanocomposite solder showed a significantly better reduction in the contact angle than the non-reinforced SAC305 solder. As shown in Figure 7 and Figure 8, the best contact angle recorded was 24.1° for the non-reinforced SAC305 solder and 18.6° for the reinforced SAC305 nanocomposite solder reflowed for 60 s. There was a significant decrease in the contact angle by 8.37% in the non-reinforced SAC305 solder and 14.29% in the reinforced SAC305 nanocomposite solder. Based on these results, it shows that, apparently, the reinforced SAC305 nanocomposite solder contributes significantly to a reduction in the contact angle. This also means that reinforcement of the SAC305 solder with TiO_2_ and Al_2_O_3_ nanoparticles functionally worked to enhance the wettability of the SAC305 solder. These results are better compared to the investigation by Tsao et al., 2010, which found that the wettability improved when reinforcing the solder with 0.50 wt.% Al_2_O_3_ nanoparticles, with a minimum contact angle of 28.9° being achieved. Research by [42] discovered an average contact angle of 32.7° with the introduction of TiO_2_ nanoparticles, which is higher than in this study. This SAC305 nanocomposite solder was also found to offer a lower contact angle compared with other types of solders: Sn-2.0Ag-0.5Cu-1.0Bi (35.6°), SAC105-2Sb-4.4In-0.3Bi (34.7°), In-Bi-Zn (24°), and In-Bi-Sn (35°) [43,44,45,46]. A solder alloy is considered to be very good when the contact angle is 0 < θ < 20° and considered good in the range of 20 < θ < 40° [2]. However, a longer reflow time up to 80 s did slightly increase the contact angle of both the non-reinforced SAC305 solder (27.4°) and the reinforced SAC305 nanocomposite solder (23.6°). The SAC305 nanocomposite solder nevertheless possessed a better contact angle compared with the non-reinforced SAC305 solder and thus enhanced the wettability performance.

The increase in the spreading area and reduction in the contact angle with increasing reflow time could be attributed to the low surface tension between the molten solder and the Cu substrate. Surface tension is one of the main factors that can determine the wetting behavior of a solder [45]. The surface tension of the reinforced SAC305 nanocomposite solder was observed to be lower than that of the SAC305 solder when reinforced with TiO_2_ and Al_2_O_3_ nanoparticles. The low surface tension decreases the boundary tension between molten solder and solid substrate, thus decreasing the contact angle, thanks to the high surface-active characteristics of both TiO_2_ and Al_2_O_3_ nanoparticles. TiO_2_ and Al_2_O_3_ nanoparticles in the solder could probably act as agents to reduce the surface tension between the molten solder and the Cu substrate [9,17,18]. This phenomenon causes increments in the spreading area and reductions in the contact angle when the surface tension between molten solder and Cu substrate is lowered. When the surface tension of the molten solder is lowered, the force of attraction between the molten solder molecules and the atoms in the solid Cu substrate becomes stronger, and hence the molten solder starts to spread easily on the solid surface of the Cu substrate [45,47]. TiO_2_ nanoparticles influence the wettability performance of nanocomposite solders by decreasing the wetting time and increasing the wetting force [17]. A study concluded that the adsorption of nanoparticles at the interface between molten solder and substrate reduced the surface energy of the molten solder and thus improved the wettability when micro- or nanoparticles were introduced to lead-free solder alloys [48]. TiO_2_ and Al_2_O_3_ nanoparticles were adsorbed and distributed along the grain boundary site, which has higher energy, thus influencing the wettability. Similar findings by [49] showed that the wettability of a solder was improved by the effective surface energy of the solder when reinforced with nanoparticles. In other words, the lower the surface tension of the molten solder on the substrate, the better the wetting properties of the solder alloy will be. The TiO_2_ and Al_2_O_3_ nanoparticles spread at the interface between the solder and Cu substrate, acting as barriers to prevent the diffusion of Cu atoms from the Cu substrate towards Sn atoms in the solder during soldering.

IMC layers that could be grown at the interface between solder and substrate are Cu_6_Sn_5_ and Cu_3_Sn upon soldering at temperatures lower than 350 °C [50]. Cu_6_Sn_5_ IMCs are formed by the dissolution of Cu and a chemical reaction between Cu and Sn atoms at the interface. The growth mechanism of IMCs occurs through diffusion at the interface on the surface between the solder and the substrate during reflow and aging at different temperatures [51]. In the diffusion between this solder and Cu, the development of the IMCs occurs in two ways. The first way is inter-diffusion and the second is an interfacial reaction between the solder and the substrate. It was reported that Cu is superior in the Sn–Cu inter-diffusion process, and Cu diffuses towards Sn at a higher rate because Cu atoms have a lower atomic radius (0.128 nm) than Sn atoms (0.141 nm) [52,53]. In the second way, the interfacial reaction, IMCs are said to form by a chemical reaction between Sn and Cu on the solder/Cu surfaces. These two ways influence each other during the formation and growth of IMCs between solder and substrate. The diffusion allows the Cu atoms to react with the Sn atoms, while the chemical reaction causes the atoms (Cu and Sn) to form the IMCs [8].

**Figure 9:**
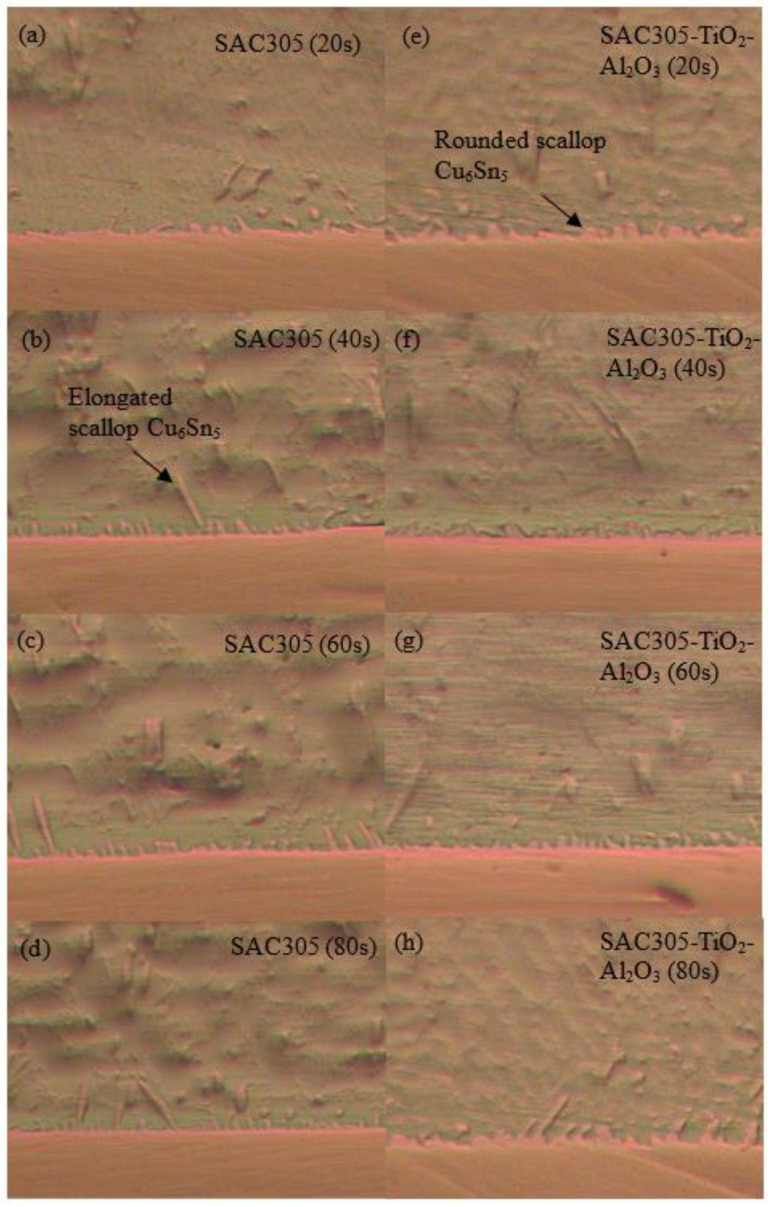
Optical micrographs of (**a**–**d**) SAC305 solder and (**e**–**h**) SAC305 nanocomposite solder under different reflow times.

**Figure 10:**
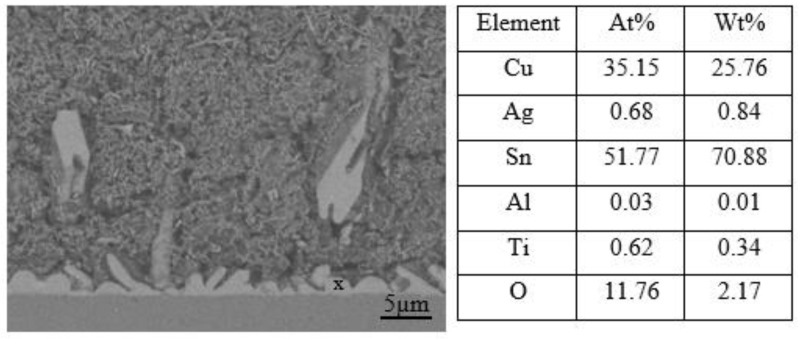
Scanning electron micrograph of SAC305 nanocomposite solder and EDX analysis for point X.

Optical micrographs of the cross-section of the SAC305 nanocomposite solder compared with the SAC305 solder at the interface between solder and substrate subjected to reflow times of 20, 40, 60, and 80 s are shown in Figure 9a–h. The IMC layers of the samples are present in the elongated and rounded scallop shape of Cu_6_Sn_5_ with a thin layer of Cu_3_Sn. Cu atoms from the Cu substrates diffuse and react with Sn atoms in the molten solder, thus forming Cu_6_Sn_5_, followed by Cu_3_Sn during soldering. The IMC layer thickness is substantially increased by increasing the reflow time. The thickness of the Cu_6_Sn_5_ IMC layer was found to decrease in this study when reflowed for 60 s. The decrease in the Cu_6_Sn_5_ IMC layer was due to the formation of a thin layer of Cu_3_Sn. The growth rate and dissolution rate of IMCs decreased upon increasing soldering time [54]. The growth rate of IMCs reduced after some time as the formation of IMCs was controlled by a diffusion reaction and a high rate of growth [55]. Hence, the IMCs’ rate of growth decreased when reaching a specific thickness, controlled by the grain boundary diffusion mechanism. TiO_2_ and Al_2_O_3_ nanoparticles exposed at the molten solder tend to disperse and attract each other by Van der Waals forces along the grain boundary and then decrease the growth kinetics of the atoms, thus refining the grain size [12,56,57]. The addition of TiO_2_ and Al_2_O_3_ nanoparticles to SAC305 solder will hinder the rate of diffusion of Cu atoms from the Cu substrate to the molten solder and thus restrict the growth of IMC layers. In addition, the growth of Cu_6_Sn_5_ was restricted by the Cu_3_Sn phase, as only a small number of Cu atoms could diffuse to the Cu_6_Sn_5_ phase from the Cu substrate. In comparison, the IMC layer of SAC305 nanocomposite solder containing 0.50 wt.% of TiO_2_ and Al_2_O_3_ nanoparticles is thinner than that of the SAC305 solder. The addition of TiO_2_ and Al_2_O_3_ nanoparticles to the SAC305 solder inhibits the growth rate of the IMC layer and reduces grain size during reflow and somehow enhances the reliability of the micro solder joints [14,58,59]. The addition of TiO_2_ nanoparticles to SAC305 was found to greatly improve the reliability of a solder by restricting the growth of IMCs at the interface between the solder and the Cu substrate, enhancing wettability, and shortening the length of IMCs in the solder matrix [17]. Inhibition or suppression of the IMC layer was attributed to the adsorption of TiO_2_ and Al_2_O_3_ nanoparticles at the interface between the solder and the Cu substrate during solidification, which reduces the surface energy of Cu_6_Sn_5_ grains and thus decelerates the growth rate of the IMC layer [20,59,60]. The adsorption effect of TiO_2_ and Al_2_O_3_ nanoparticles not only restricts the growth of the IMC layer but also serves as a diffusion barrier at the interface between the molten solder and the Cu substrate. However, the thickness of the IMC layer could increase due to the excessive addition of TiO_2_ and Al_2_O_3_ nanoparticles, which can later degrade the reliability of solder joints. Figure 10 confirms the presence of TiO_2_ and Al_2_O_3_ nanoparticles at the IMC/solder interface (point X) and the elemental composition is summarized.

**Figure 11:**
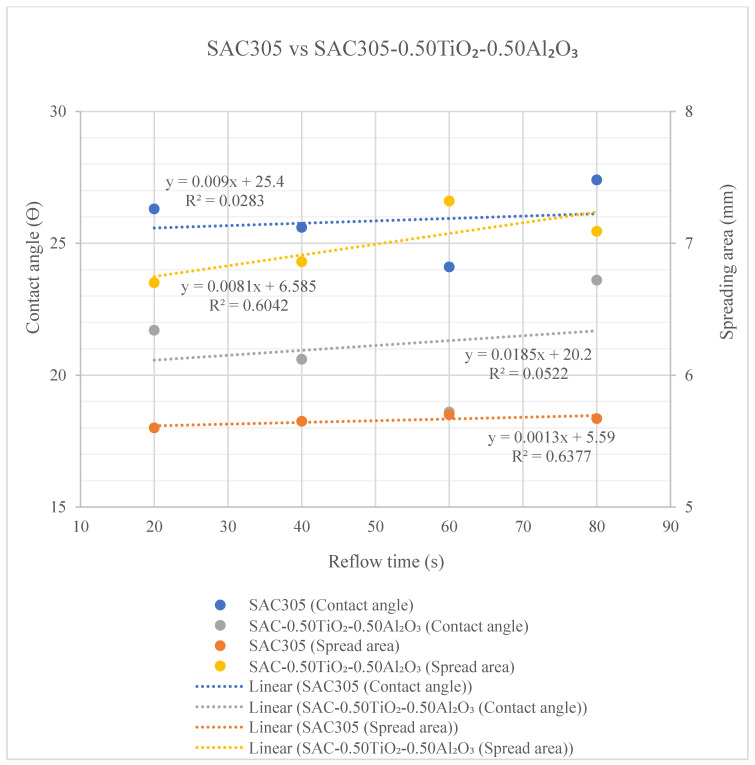
Contact angle and spreading area for non-reinforced SAC305 solder and reinforced SAC305 nanocomposite solder at different reflow times.

Correlations between contact angle and spreading area for both the non-reinforced SAC305 solder and the reinforced SAC305 nanocomposite solder at different reflow times are shown in Figure 11. Shown in the figure below the representation of the contact angle are non-reinforced SAC305 solder (blue) and reinforced SAC305 nanocomposite solder (grey). As for the spreading area, the trend lines are shown for non-reinforced SAC305 solder (orange) and for reinforced SAC305 nanocomposite solder (yellow). From these results, it can be interpreted that the contact angle is perpendicular to the spreading area. As the reflow time increases up to 60 s, the contact angle decreases while the spreading area increases. The reduction in contact angle and increment in spreading could be attributed to the increase in the flux reaction rate [47]. From the trend line data shown in the graph below, R^2^ measures how well the variation in the data fits the linear model that ranges between 0 and 1 (i.e., 0 to 100%), meaning the larger the R^2^, the better the model explains the observed data. The R^2^ value for the contact angle of non-reinforced SAC305 (blue) is 0.0283 (0.3%) and that of reinforced SAC305 nanocomposite solder (grey) is 0.052 (0.50%), and this means that the data for reinforced SAC305 nanocomposite solder are best fitted to the linear trend line. An opposite trend was observed for R^2^ values for spreading area; here the value of reinforced SAC305 nanocomposite solder (yellow) is 0.6042 (60.42%), not well fitted, and that of non-reinforced SAC305 (orange) is 0.6377 (63.77%) due to larger differences in spreading area measured for reinforced SAC305 nanocomposite solder. The spreading rate of the SAC305 solder was significantly greater after the addition of TiO_2_ and Al_2_O_3_ nanoparticles, with a larger area and a smaller contact angle.

## 4. Conclusions

The wettability performance of non-reinforced SAC305 solder and reinforced SAC305 nanocomposite solder was investigated. The melting point of SAC305 nanocomposite solder increased slightly due to the high melting temperatures of both TiO_2_ and Al_2_O_3_ nanoparticles. The wettability was measured based on the contact angle and spreading area under different reflow time ranges of 20–80 s. The wettability performance improved with lower contact angles and larger spreading areas when reinforced with 0.50 wt.% of TiO_2_ and Al_2_O_3_ nanoparticles reflowed up to 60 s. As the reflow time increased, the surface tension between the solder and the Cu substrate was found to decrease and improve the ability of molten solder to spread easily with lower contact angles. The correlation between contact angle and spreading area was evaluated; the contact angle is perpendicular to the spreading area. Elongated and round scallop IMCs were observed to form at the interface between the solder and the Cu substrate. A higher wettability with lower contact angle and larger spreading area was achieved when reinforcing SAC305 solder reflowed for 60 s with TiO_2_ and Al_2_O_3_ nanoparticles.

## Data Availability

The data presented in this study are available upon reasonable request to the corresponding author.
